# Quantitative Assessment of Low-Dose Photodynamic Therapy Effects on Diabetic Wound Healing Using Raman Spectroscopy

**DOI:** 10.3390/pharmaceutics15020595

**Published:** 2023-02-10

**Authors:** Hala Zuhayri, Alice A. Samarinova, Alexey V. Borisov, David A. Lopez Guardado, Houssain Baalbaki, Natalya A. Krivova, Yury V. Kistenev

**Affiliations:** Laboratory of Laser Molecular Imaging and Machine Learning, Tomsk State University, Lenin Ave. 36, Tomsk 634050, Russia

**Keywords:** wound healing, diabetes, low dose photodynamic therapy, Raman spectroscopy, principal component analysis, Mahalanobis distance

## Abstract

One of challenges that faces diabetes is the wound healing process. The delayed diabetic wound healing is caused by a complicated molecular mechanism involving numerous physiological variables. Low-dose photodynamic therapy (LDPDT) provides excellent results in rejuvenation and wound healing. In this study, the LDPDT effect on diabetic wounds in mice was studied using two photosensitizers, 5-aminolevulinic acid and methylene blue, and two laser dose expositions of 1 J/cm^2^ and 4 J/cm^2^ by Raman spectroscopy (RS). The latter was used as a noninvasive method, providing specific information about tissue state based on the fundamental vibrational modes of its molecular components. RS allows high spatial resolution acquisition of biochemical and structural information through the generation of point spectra or spectral images. An approach to in vivo quantitative assessment of diabetic wound healing state was developed. This approach is based on an application of the principal component analysis combined with the Mahalanobis metrics to skin Raman spectra, in particular, intensities of the amide I and CH_2_ bands.

## 1. Introduction

Diabetes mellitus (DM) has become a global burden of over 537 million patients worldwide [1]. Diabetes is not only a huge health burden, but it also causes a considerable cost on society, economies, and health care systems. DM is a chronic, metabolic disease characterized by compromised insulin secretion, resistance to insulin action, or both, leading to hyperglycemia [2,3]. In fact, as a consequence of hyperglycemia, diabetic patients suffer from improper functioning of heart, blood vessels, eyes, kidneys, and nerves. The impairment of self-repairing abilities is one of diabetes’ major complications [4].

DM is one of the challenges of the wound healing process [5]. Normal wound healing is a dynamic and complex physiological process involving the following overlapping phases: hemostasis, inflammation, proliferation, and remodeling [6,7]. The wound healing begins with hemostasis achieved through vasoconstriction and platelet-mediated activation of the intrinsic clotting cascade, ending in formation of a fibrin clot [6,8]. The fibrin-plug formation is overlapped with the inflammatory phase, which is characterized by lymphocyte, macrophage, and neutrophil infiltration of the wound site to eliminate pathogens, bacteria, and cellular debris [5,9]. Then, the proliferative stage begins with the formation of new tissue, blood vessels (angiogenesis), and matrix construction to fill the wounded area [10]. Wound closure begins with the contraction caused by granulation tissue, keratinocyte migration, formation of extracellular matrix (ECM), and the appearance of myofibroblasts [2]. The role of the fibroblasts is wound-area reconstruction through collagen release. The final remodeling phase consists of collagen fibers’ reorganization, when collagen type III accumulating first is replaced by collagen type I, followed by the neovascularization, and then going back to the norm [11]. At this stage, the wound repair proceeds towards to the restoration of the physiologic structure of skin [12].

In diabetes, the wound healing is delayed due to the following biochemical processes. Inflammatory cytokines are present with high concentrations in the affected area, indicating the continued inflammatory response for a long period [13]. In addition to changes in growth factors, such as IGF-1 and TGFβ, insulin decreasing occurs in diabetics’ wound tissue [2,14]. Furthermore, there are many additional factors delaying the wound healing, including specific metabolic deficiencies, hypoxia caused by hemoglobin glycation, alteration of red blood cells’ membranes, and narrowing blood vessels [5,15]. The oxygen supply decreases in wounds, which, in turn, delays the healing. 

Oxidative stress is caused by the increasing reactive oxygen species (ROSs) production. Oxidative stress occurs at the molecular level as a cellular event when ROS increasing overwhelms the antioxidant system’s defense capabilities. It varies in intensity, cell location, and time scale, i.e., acutely or chronically [16,17]. ROSs play a key role in the wound healing process. Increased ROS levels in chronic wounds may have a variety of deleterious effects. Oxidative stress may influence diabetic wound healing through skin injury, neuropathy, ischemic lesion, and topical infections [18]. 

Low-dose photodynamic therapy (LDPDT) is widely used in dermatology and produced worthwhile results in skin rejuvenation and wound healing [19,20,21]. LDPDT is based on a photosensitizer (PS) accumulation in tissues, following by irradiation with an appropriate wavelength light source, producing ROSs [22,23]. 5-aminolevulinic acid (5-ALA) and methylene blue (MB) are considered to be the most effective PSs [24,25,26]. The tissue response to photonic energy follows the Arndt–Schultz pattern, which is a suitable model to describe dose-dependent effects of a low-level laser therapy. A small stimulus may have no biological effect, a moderate stimulus may have a biostimulatory effect, and a large stimulus may have a bioinhibitory or even cytotoxic effect [27]. In accordance with the Arndt–Schultz pattern, it was found that the dose of 1 J/cm^2^ is close to the lower threshold of the biological response. Then, the curve begins to rise, which means an increase in the biological response with a laser light dose increase. The curve reaches the upper threshold around the laser light dose of 4 J/cm^2^, which is considered in the most effective range. After that, the biological response begins to decrease, indicating a bioinhibitory effect. Previous studies have found that the optimal light doses for LPDT are practically the same and should be in the 1–5 J/cm^2^ range [28,29,30,31].

Raman spectroscopy (RS) is a non-invasive and non-destructive method of chemical analysis with a high molecular specificity widely used for medical diagnostics and assessment of biophysical processes [32,33]. RS is a vibrational spectroscopic technique based on the registration of light inelastic scattering by molecules. The latter is caused by the excitation of a molecule by incident photons to a virtual energy state and the resultant energy loss (Stokes) or gain (anti-Stokes) occurs because of the interaction of light with vibrational modes associated with molecular chemical bonds. This shift in energy is associated with discrete vibrational modes of polarizable molecules, and thus, a sample biochemical composition can be discovered [34]. RS has been demonstrated to be a powerful analytical technique in the study of biological materials, and it allows for high spatial resolution acquisition of biochemical and structural information through the generation of point spectra or spectral images [35]. RS allows for in vivo tissue analysis, including skin [36,37]. The biological samples have a unique biological “fingerprints”, allowing understanding of the specimen, chemical structure, and composition [34]. The informative ranges in the Raman spectrum of a typical biological sample are as follows. The spectral range of 1500–1700 cm^−1^ is associated with bond vibrations of proteins. Phosphate groups’ spectral peaks are near 980, 1080, and 1240 cm^−1^. Carbohydrates are in the range of 470–1200 cm^−1^. High-frequency bond vibrations associated with CH, NH, and OH stretching in lipids and proteins can also be observed in the range of 2700–3500 cm^−1^ [38,39].

In oncology studies, RS was used to diagnose prostate, pancreatic, breast, and oral cancers [40]. In neurodegenerative diseases studies [41], RS was used to diagnose Alzheimer’s [42] and Parkinson’s [43] diseases. RS was applied in the analysis of metabolic syndrome [44], and metabolic changes during drug treatment in live cancer cells and tissues [45]. Correlations between Raman spectra in vivo data and X-ray microanalysis in vitro data in skin studies were established [46]. It is also worth noting the differences presented between the in vivo and in vitro Raman spectra of human skin. However, both forms represent a reliable technique to describe the information about the lipid and natural moisturizing factor content of the stratum corneum [47]. This evidence allows more profound research on the characterization of human skin, and, therefore, the research of wound healing process. So far, the description of such a process has been achieved from the study of skin wounds in rodents. It was shown how the RS technique in vivo can provide insight into the status of normally healing wounds [48]. The ability to study the burn-induced conformational changes of collagen using RS was confirmed [49]. Furthermore, RS has allowed for the development of other techniques, such as depth-sensitive RS, to perform depth-sensitive measurements [50]. In previous studies with RS, the difficulties facing wound healing, such as diabetes complications, were not studied, nor was a quantitative assessment and comparison of wound healing treatment using LDPDT with different parameters.

The aim of this work is to develop a quantitative method for in vivo assessment of the wound healing process in diabetics by RS, and to study the effect of LDPDT using topical application of two different photosensitizers: 5-ALA and MB and two laser doses of 1 J/cm^2^ and 4 J/cm^2^.

## 2. Materials and Methods

### 2.1. Type 1 Diabetes Model 

Fifty laboratory CD1 six-seven-week-old male mice weighing 25–30 g obtained from the Department of Experimental Biological Models of the Research Institute of Pharmacology, TSC SB RAS, Tomsk, Russia were used in the study. The experimental protocol of this research was approved by the Bioethical Committee of Tomsk State University, Tomsk, Russia (Protocol No.4, 10.02.2021, registration No. 6). Before the experiments, the mice were kept for 7 days in standard conditions of a conventional vivarium with free access to food and water, and a 12/12 light regime, in a ventilated room at a temperature of 20 ± 2 °C and a humidity of 60%. All mice were weighed and randomly divided into control and experimental groups, each group consisted of 25 mice. For induction of type 1 diabetes mellitus in mice, the standard protocol of low dose Streptozotocin (STZ) injections was implemented [51]. Diabetic model was conducted in two weeks. The first stage was the treatment of mice with STZ for 5 days. In this stage, STZ (40 mg/kg) was injected daily after fasting 6 h, and water was replaced with 10% sucrose water. After completing the five-day STZ protocol, the 10% sucrose water was replaced by pure water.

The second stage was measuring the blood glucose level via a tail-vein using the “One Touch Flex” blood glucose monitoring system (Switzerland). For later-stage diabetes, after 15 and 25 days of last STZ injection, the process of blood glucose measurement was repeated. The results are presented in Table 1. The mice were weighed in all stages.

### 2.2. In Vivo Wound Model

**Figure 1:**
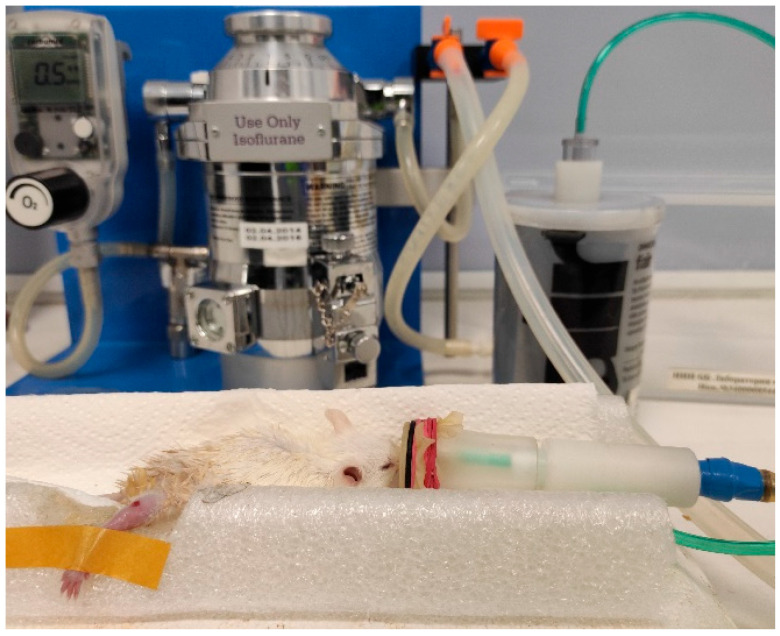
A mouse anesthetized by isoflurane, using the Ugo Basile gas anesthesia system (Italy).

Full-thickness wounds were created surgically on hind limbs as follows. The mice were anesthetized by isoflurane with concentration of about 3–4% for 10–15 min, using the Ugo Basile gas anesthesia system (Italy). After that, the mouse was put on a base with a mask on the nose with 0.5–1.5% isoflurane flow, as shown in Figure 1. The wound area was preliminarily depilated by Veet cream (France) and sterilized using chlorhexidine 20%. The right hind limb skin was folded and raised using forceps by medical scissors. The skin layers were completely removed and full-thickness excisional circular wounds (5 mm diameter) were created. The day of wound formation was denoted as day 0. 

### 2.3. Wound Healing Assay

The wound area was estimated by measuring its larger A and minor B diameters by a digital caliper. The accuracy of the latter was 0.02 mm. This value makes an insignificant contribution to the measurement error and was not taken into account. The wound area S was calculated using the formula:

S = (A × B × π)/4.

### 2.4. Low Dose Photodynamic Therapy Protocol

**Figure 2:**
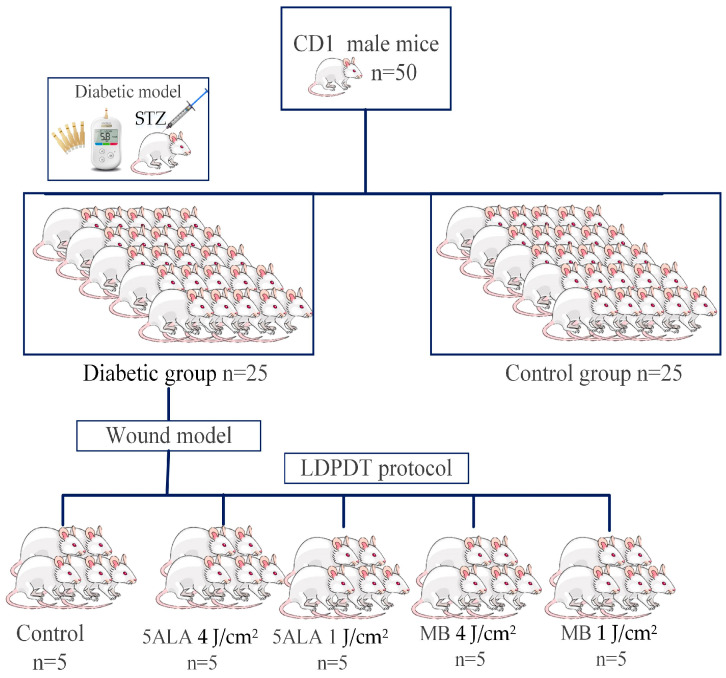
The experimental research design.

While the mouse was under the influence of isoflurane, the photosensitizers 5-ALA and MB in saline solution were topically administered for 30 min on a wound, dripping 0.5–1 mL of PS solution directly on the wound. The wounds were irradiated by a CW AlGalnP laser (λ = 630 nm, *p* = 5 mW) with two doses: 1 J/cm^2^ or 4 J/cm^2^ for 3 min 45 s and 15 min, respectively. These doses were chosen, according to the Arndt–Schultz’s pattern of biological response [31], and they were used in the authors’ previous studies [7,21]. The diabetic mice were randomly divided into five groups; each group included 5 mice: the control group, LDPDT-5-ALA/1 J/cm^2^, LDPDT-MB/1 J/cm^2^, LDPDT-5-ALA/4 J/cm^2^, and LDPDT-MB/4 J/cm^2^ groups. The LDPDT procedure was conducted once immediately after wound formation. The experimental research design is presented in Figure 2.

### 2.5. Raman Spectroscopy

**Figure 3:**
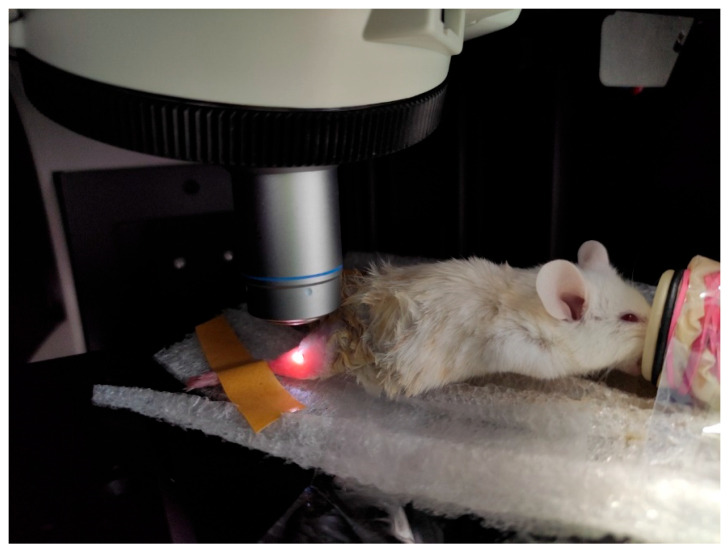
The mouse in the working field of the confocal Raman spectrometer.

Raman spectra were measured by a Renishaw confocal Raman spectrometer (UK) with a spectral resolution of 1 cm^−1^, and spatial resolutions of 1 μm (horizontal) and <2 μm (vertical). A 785 nm diode laser with an output of 120 mW was used for Raman scattering excitation. An isoflurane-anesthetized mouse was placed on a foam pad. The leg with the wound was additionally fixed in the desired position. The leg was fixed in such a way that the surface of the wound was close to perpendicular to the incident laser radiation. After that, the mouse was placed in the working field of the spectrometer, as shown in Figure 3. The laser beam was focused on the sample through a 50 × lens. The Raman spectra wave-number was calibrated using a silicone sample before each test. 

**Figure 4:**
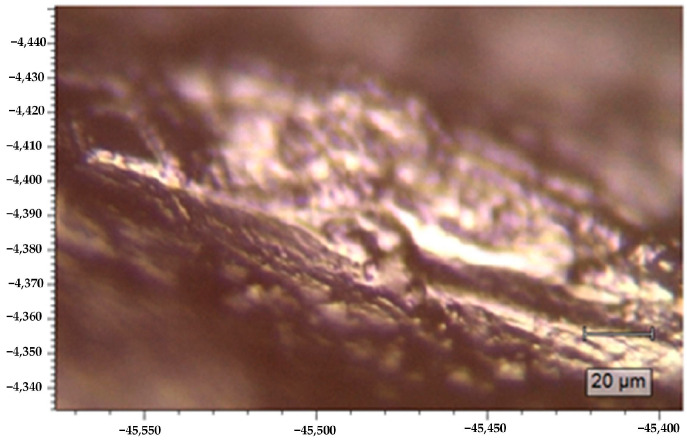
A tissue image on the Raman spectrometer camera screen.

In the Raman spectrometer, the focus of laser radiation beam coincides with the focus of the camera. The motorized stage on which the mouse was placed was moved until a clear image of the tissue was seen in the camera (Figure 4).

After focusing on the selected point of the wound surface, the recording of spectral data began. Raman spectra were collected from 10 different spatial points superficially for each wound with a 10 s integration time and 3 accumulations. Spectra with a high cosmic noise were discarded. 

Experiments were conducted on 3 mice out of 5 in each group, in order to maintain constant statistics throughout the duration of the experiments, and thus it is possible to replace mice whose condition worsens or dies due to anesthesia during the measurement operations. The measurements were conducted on days 1, 3, 7, and 14 after wound creation. In every experimental day, 30 Raman spectra were measured from 3 mice (10 spectra for every mouse at different points in the wound). For each group, 120 spectra for all control days were measured in the spectral range of 100–3500 cm^−1^. Healthy skin Raman spectra were measured in each group by 30 spectra (10 spectra for every mouse). For all groups, on all days were measured 750 spectra.

### 2.6. Data Preprocessing

**Figure 5:**
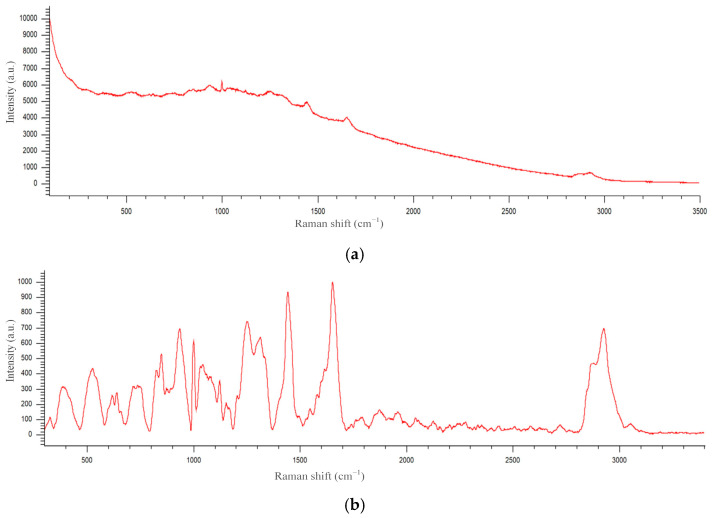
An example of a Raman spectrum before (**a**) and after (**b**) background subtraction.

Tissue Raman spectrum usually contains a fluorescence background. Although near-infrared excitation (785 nm) provides a low fluorescence background, it is still necessary to remove it due to its potential effect on the subsequent analysis accuracy. The background baseline was fitted by a polynomial of order 18 and subtracted using Renshaw WiRE^TM^ software (see Figure 5). Then, normalization was performed to bring all the spectra to the same interpretable scale. The spectra in each group were presented in terms of “mean ± standard deviation”.

### 2.7. Statistical Analysis

Principal Component Analysis (PCA) was applied to Raman spectra to explore the factors thought to be important in wound healing [52]. PCA uses orthogonal transformation of potentially correlated initial coordinates to linearly uncorrelated principal components (PC). PCA was carried out using the Matlab software.

Mahalanobis distance ($d_{M}$) was used to assess a state of skin in relation to its ‘reference’ state. In this study, the latter corresponds to the healthy skin. The Mahalanobis distance between the *i* and *j* data samples is determined as follows:

(1) $$d_{M}\left({\vec{x}}_{i},{\vec{x}}_{j}\right)=\;d{\left({\vec{x}}_{i},{\vec{x}}_{j}\right)}^{T}C^{-1}d\left({\vec{x}}_{i},{\vec{x}}_{j}\right)$$

where ${\vec{x}}_{i}$ is a Raman spectrum of the skin sample belonging to a studied state, ${\vec{x}}_{j}$ is the same for healthy skin, $C^{-1}$ is the variance–covariance matrix of a set $S_{H}$ of healthy skin Raman spectra. Let us denote by S a section of Raman spectra of skin belonging to a studied state. Then, the difference of the sets of Raman spectra of skin belonging to a studied state and to the reference state $d\left(S,S_{H}\right)$ can be calculated as follows:

(2a) $$d\left({\vec{x}}_{i},S_{H}\right)=\frac{1}{N_{S_{H}}}\;\;\overset{N_{S_{H}}}{\underset{j}{\sum }}d_{M}\left({\vec{x}}_{i},{\vec{x}}_{j}\right),$$

(2b) $$d\left(S,S_{H}\right)=\frac{1}{N_{S}}\overset{N_{S}}{\underset{i}{\sum }}d\left({\vec{x}}_{i},S_{H}\right),$$

where ${\vec{x}}_{i}$, ${\vec{x}}_{j}$ are the objects from sets *S*, *S_H_*, and $N_{S}$,$\;N_{S_{H}}$ are the sizes of the sets *S*, $S_{H}$, respectively. The Student’s *t*-test was used to determine if there is a significant difference between the two independent datasets. The level of significance was set at *p* < 0.05.

## 3. Results

### 3.1. Characteristics of the Studied Mice

The following characteristics of studied mice were measured: weight, the blood glucose level, and water consumption, as presented in Table 1. 

Water consumption difference between the control and diabetic groups was measured. During a week, the control group consumed (200 + 20) mL, while the diabetic group consumed (500 ± 75) mL.

### 3.2. Wound Area Analysis

**Figure 6:**
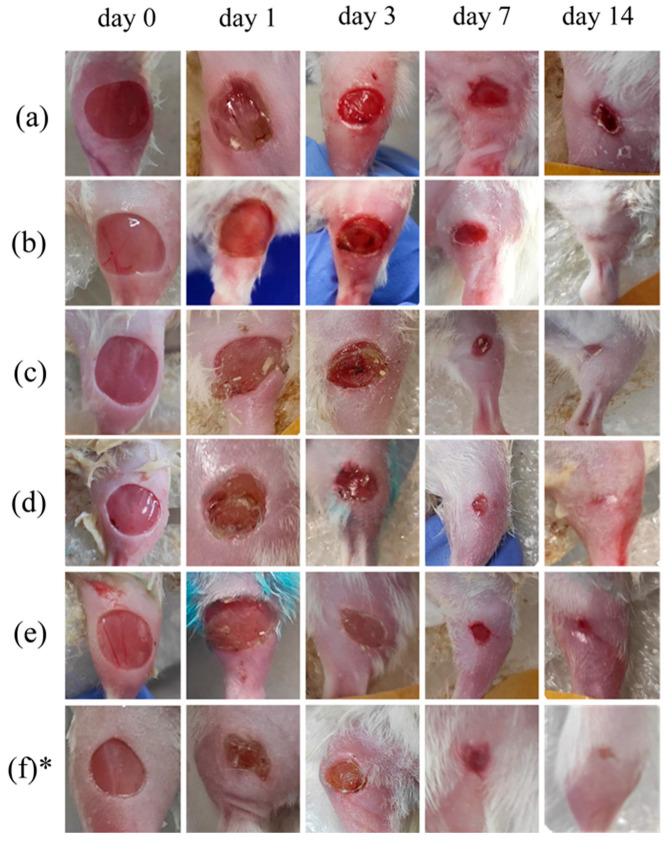
Digital photos of the wound healing progression from day 0 to day 14: (**a**) control group, (**b**) 5-ALA 4 J/cm^2^ group, (**c**) 5-ALA 1 J/cm^2^ group, (**d**) MB 4 J/cm^2^ group, (**e**) MB 1 J/cm^2^ group, (**f**) healthy control without diabetes. * This line of pictures was adapted from [6] under CC BY 4.0 license. 2022, MDPI).

The wound area was monitored at different time points on days 1, 3, 7, and 14. Wound digital images were captured as presented in Figure 6. A comparison was conducted between the control, LDPDT groups, and the healthy control group without diabetes that was in an early series of wound healing experiments. The enhancement of wound healing becomes apparent from the third day and evident from the seventh day after LDPDT. The 4 J/cm^2^ LDPDT groups exhibited a statistically significant effect on wound closure on days 3, 7, and 14 when compared to the control group.

The wound percentage was calculated as the value of a wound area to the initial one by the following equation:

[(S_0_ − S)/S_0_] × 100, (3)

where S_0_ is the area of the wound at day 0 and S is the area of the current wound.

**Figure 7:**
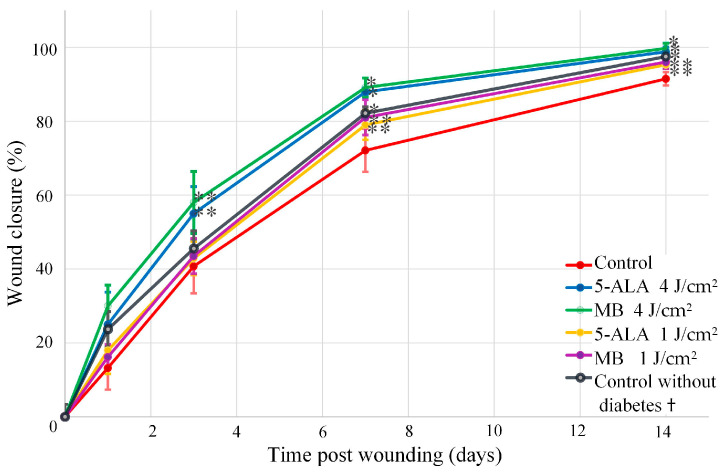
Wound healing progression in the following groups: red line—control, blue line—5-ALA 4 J/cm^2^, green line—MB 4 J/cm^2^, yellow line—5-ALA 1 J/cm^2^, purple line—MB 4 J/cm^2^, black line—control without diabetes. (The number of mice was equal to 3 for each group), * *p*-value < 0.05, ** *p*-value < 0.1. † The wound areas of control without diabetes were taken from a previous study of the authors [6].

Different wound closure was observed in LDPDT groups compared to the control. The wound closure percentage was presented in Figure 7 in terms of “mean ± standard deviation”. These results reveal the gradual closure of the wound and the difference between studied groups. The Student’s *t*-test was used to check observed differences statistically, and *p*-value was calculated for all days in comparison with the control. 

### 3.3. Raman Spectra of Healthy Skin

**Figure 8:**
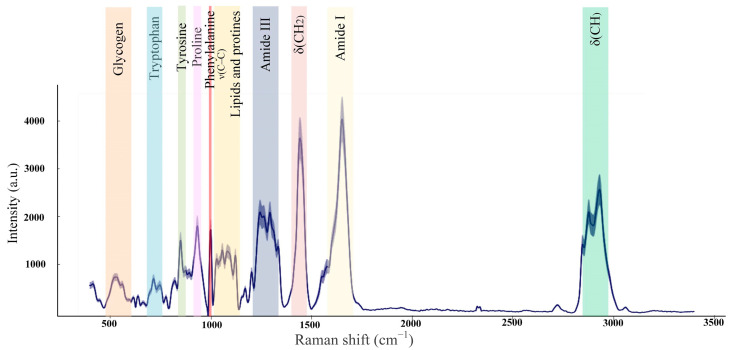
Mice healthy skin average Raman spectra (the number of processed Raman spectra was equal to 20).

The healthy skin averaged Raman spectra are presented in Figure 8. In the 1200–1700 cm^−1^ range, the amide bands associated with amide bond vibrations in polypeptide chains are observed [53]. The amide I band is dominated by the ν(C=O) vibrations, while the amide III band by the ν(C–N) and δ(N–H) vibrations [54]. In the Raman spectrum of healthy skin, the peak of the amide I band was identified near 1658 cm^−1^, which primarily corresponds to collagen type I. Within the amide III band, two peaks of 1247 cm^−1^ and 1270 cm^−1^ are associated with proline-rich (non-polar) and proline-poor (polar) spectral fingerprints of the collagen [55]. The peak 1445 cm^–1^ is dominated by the vibrations of CH_2_ bending, which indicate the total dermal matrix protein content [49,56]. In the 1050–1150 cm^−1^ spectral range, the peaks are mainly assigned to the ν(C–C) modes associated with lipid components [57]. Spectral features in the 450–1050 cm^−1^ spectral range were attributed to amino acids: 1003 cm^−1^ (phenylalanine), 936 cm^−1^ (proline), 877 cm^–1^ (hydroxyproline, tryptophan), 854 cm^−1^ (proline, tyrosine), and 755 cm^−1^ (tryptophan) [54,58]. In the 2730−3160 cm^−1^ spectral range, peaks correspond to the −CH stretching, which indicates relative quantities of total protein and lipid content [48,59].

### 3.4. Raman Spectra of Skin Wounds

**Figure 9:**
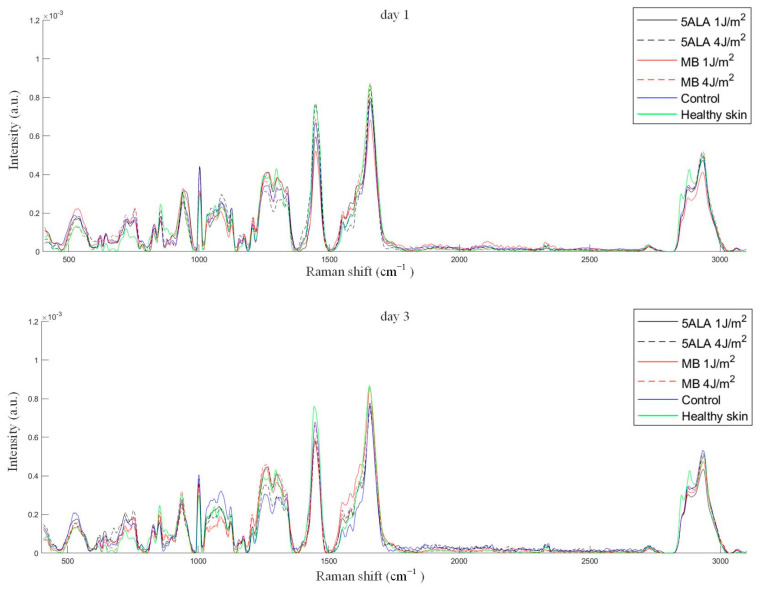

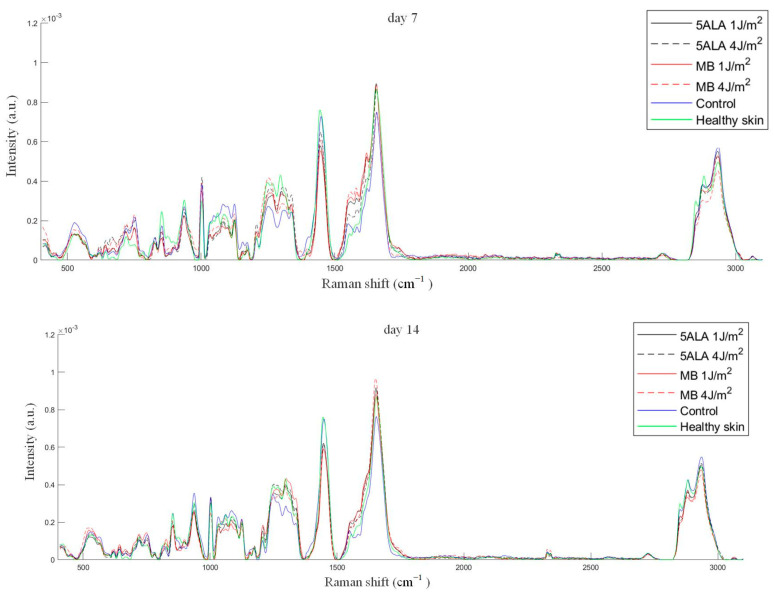
The mean Raman spectra of skin wounds in all groups on all observation days and healthy skin, black line—5ALA 1 J/cm^2^, black dashed line—5ALA 4 J/cm^2^, red line—MB 1 J/cm^2^, red dashed line—MB 4 J/cm^2^, blue line—control, green line—healthy skin (the number of processed Raman spectra was equal to 30 for each group).

The average Raman spectra of skin wounds in all groups on observation days and healthy skin are presented in Figure 9.

**Figure 10:**
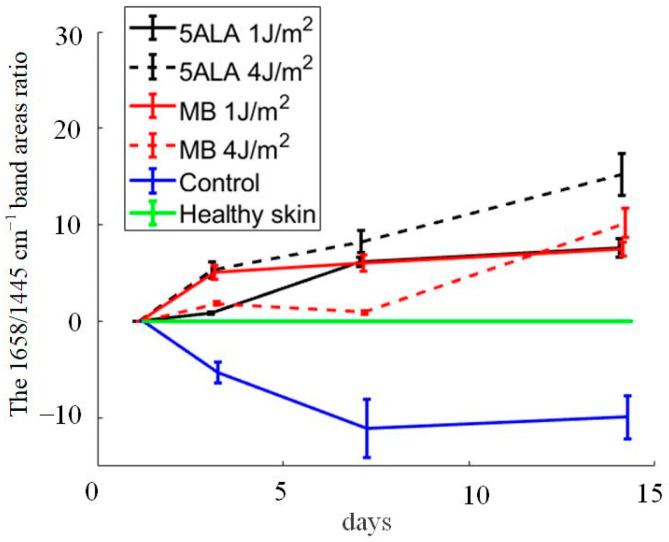
The 1658/1445 cm^−1^ band areas ratio during wound healing in the following groups: black line—5ALA 1 J/cm^2^, black dashed line—5ALA 4 J/cm^2^, red line—MB 1 J/cm^2^, red dashed line—MB 4 J/cm^2^, blue line—control, green line—healthy skin (the number of processed Raman spectra was equal to 30 for each group).

As mentioned above, the peak 1445 cm^−1^ is dominated by the CH_2_ associated with the total dermal matrix protein content, while the peak 1658 cm^−1^ is dominated by amide I associated with collagen content. The 1658/1445 cm^−1^ band areas ratio gives information on collagen content, which is an important factor in wound healing [60]. The 1658/1445 cm^−1^ band areas ratio was calculated using Gaussian peak fitting (see Figure 10). Thus, an increase in collagen content is indicated by a larger value of this ratio. The differences were observed between the control, LDPDT groups, and healthy skin on day 14. The highest collagen content was found in LDPDT 4 J/cm^2^ groups.

The Student’s *t*-test was used to check observed differences statistically, and *p*-values were calculated for all data in comparison with healthy skin, for 15 measurements for each sample, as presented in Table 2.

*p*-values for all data were lower than the selected significance level (0.05), except the control group on day 14, which seems to be caused by data random variability. It means that there are essential differences between groups. The 1658/1445 cm^−1^ band areas ratio is shown from the Table 2 to have a negative correlation with wound healing stage. The biochemical origin of this will be discussed below.

**Figure 11:**
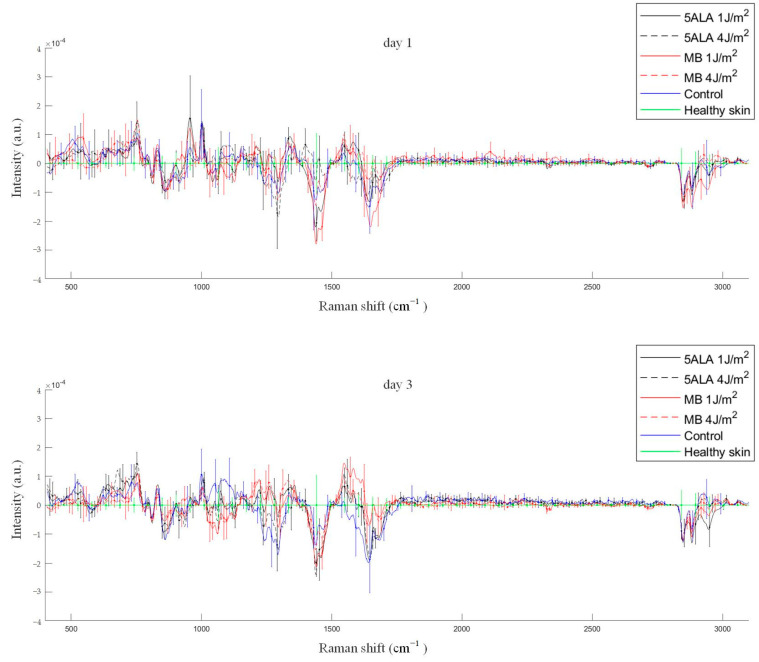

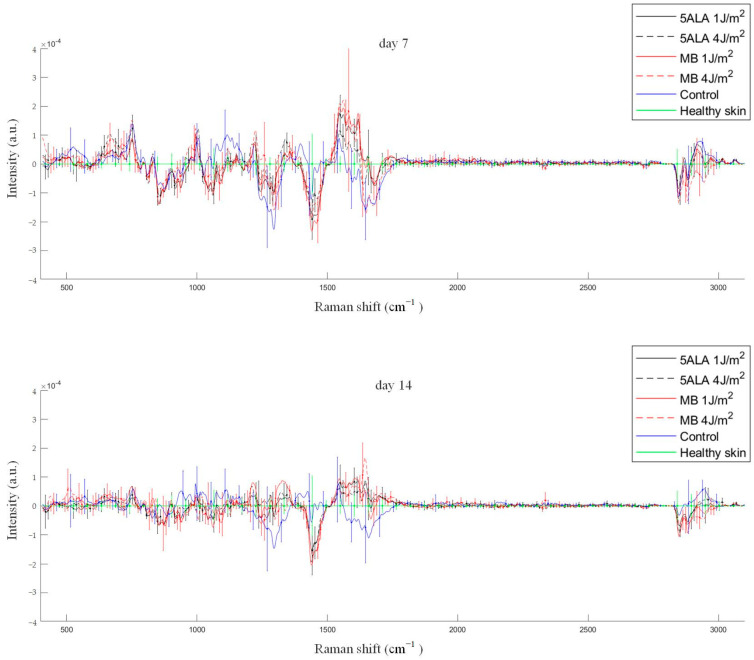
Differences between the wound Raman spectra of all groups subtracting the healthy skin mean Raman spectrum. The Raman spectra are presented in terms of mean ± StD values. Black line—5ALA 1 J/cm^2^, black dashed line—5ALA 4 J/cm^2^, red line—MB 1 J/cm^2^, red dashed line—MB 4 J/cm^2^, blue line—control, green line—healthy skin (the number of processed Raman spectra was equal to 30 for each group).

**Figure 12:**
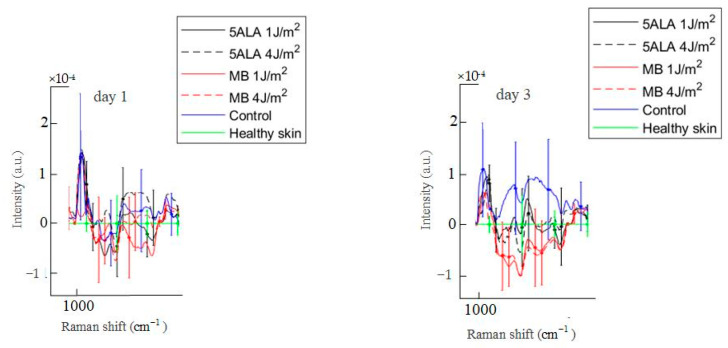

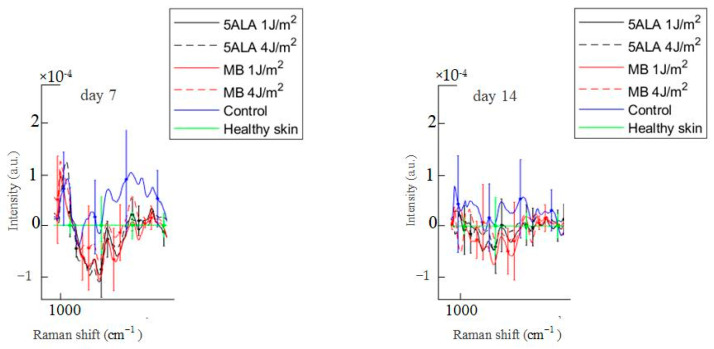
Difference between the wound Raman spectra of all groups subtracting the healthy skin mean Raman spectrum in the 1020–1140 cm^−1^ spectral range. The Raman spectra are presented in terms of mean ± StD values. Black line—5ALA 1 J/cm^2^, black dashed line—5ALA 4 J/cm^2^, red line—MB 1 J/cm^2^, red dashed line—MB 4 J/cm^2^, blue line—control, green line—healthy skin (the number of processed Raman spectra was equal to 30 for each group).

**Figure 13:**
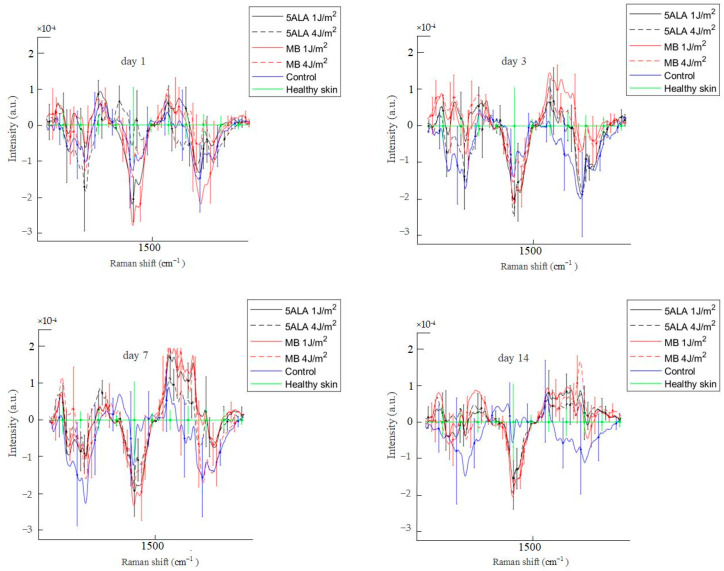
Difference between the wound Raman spectra of all groups subtracting the healthy skin mean Raman spectrum in the 1200–1750 cm^−1^ spectral range. The Raman spectra are presented in terms of mean ± StD values. Black line—5ALA 1 J/cm^2^, black dashed line—5ALA 4 J/cm^2^, red line—MB 1 J/cm^2^, red dashed line—MB 4 J/cm^2^, blue line—control, green line—healthy skin (the number of processed Raman spectra was equal to 30 for each group).

**Figure 14:**
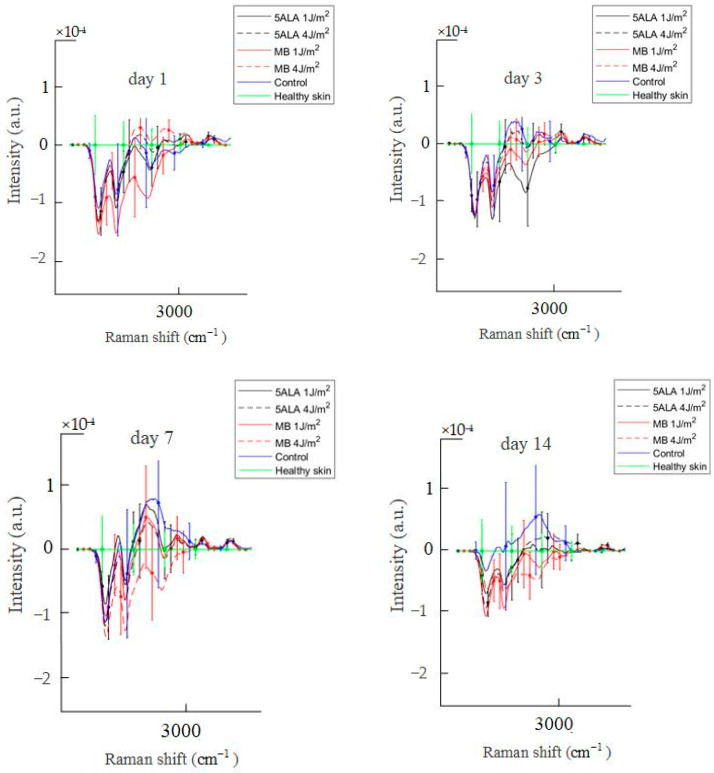
Difference between the wound Raman spectra of all groups subtracting the healthy skin mean Raman spectrum in the 2700–3000 cm^−1^ spectral range. The Raman spectra are presented in terms of mean ± StD values. Black line—5ALA 1 J/cm^2^, black dashed line—5ALA 4 J/cm^2^, red line—MB 1 J/cm^2^, red dashed line—MB 4 J/cm^2^, blue line—control, green line—healthy skin (the number of processed Raman spectra was equal to 30 for each group).

Differences in Raman spectra were estimated by subtracting the healthy skin mean Raman spectrum from skin Raman spectra for other groups (see Figure 11). In order to study the differences in more detail, the spectra were split into three spectral regions, where differences were observed: the first region corresponds to the 1020–1140 cm^−1^ range, the second region corresponds to the 1200–1750 cm^−1^ range, and the third one is in the 2700–3000 cm^−1^ range (see Figure 12, Figure 13 and Figure 14, respectively).

### 3.5. Principal Component Analysis

**Figure 15:**
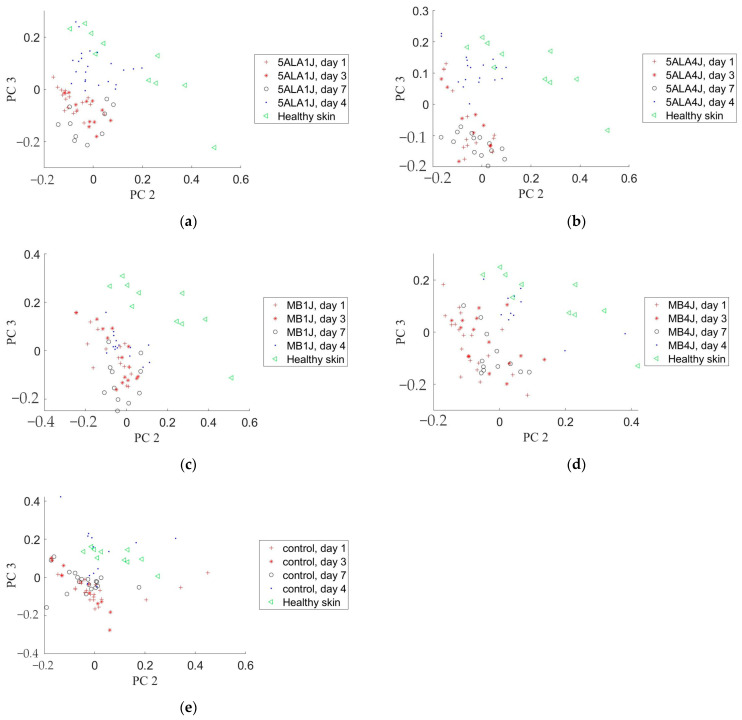
Principal component analysis of the wound and healthy skin Raman spectra in the 2800–3000 cm^−1^ spectral range on different observation days in a subspace of PC2 and PC3: (**a**) 5-ALA 1 J/cm^2^ group, (**b**) 5-ALA 4 J/cm^2^ group, (**c**) MB 1 J/cm^2^ group, (**d**) MB 4 J/cm^2^ group, (**e**) the control group.

PCA was applied to the wounds where Raman spectra related to the following spectral ranges: 600–800 cm^−1^, 1020–1140 cm^−1^, 1200–1370 cm^−1^, 1390–1500 cm^−1^, 1570–1750 cm^−1^, 1200–1750 cm^−1^, and 2800–3000 cm^−1^ to visualize the changes in skin chemical content from a point of view of distinguishing wound state in studied groups. PC2 and PC3 of Raman spectra in the range of 2800–3000 cm^−1^ were shown to be the most effective in wound healing process control. It allows distinguishing groups and days of observation (see Figure 15). For example, PC2 and PC3 of the wound Raman spectra in the range 2800–3000 cm^−1^ in 5ALA 1 J/cm^2^, 5ALA 4 J/cm^2^, and MB 4 J/cm^2^ groups at day 14 were closely clustered to the healthy skin group. The situation on days 1 and 3 is not so obvious. Similar results were not observed for other spectral sub-bands from 400 to 2800 cm^−1^.

To calculate the Mahalanobis distance, the PCs space was scaled, so that, in the PCs subspace, the healthy group was inside the area with a radius r calculated using the following formula:

(4) $$r=\sqrt{\sigma_{2}^{2}+\sigma_{3}^{2}},$$

where $\sigma_{2}$ and $\sigma_{3}$ are standard deviations for the PC2 and PC3 coordinates of the healthy group samples, respectively [61]. To simplify calculating the distance, the PCs space was normalized so that the radius r was equal to √2. After scaling PCs space, the Mahalanobis distances between distributions of healthy skin and other groups’ Raman spectra PCs were calculated using Equations (2a) and (2b). Table 3 presents the Mahalanobis distance in the PC2–PC3 space between the healthy skin and 5ALA 1 J/cm^2^, 5ALA 4 J/cm^2^, MB 1 J/cm^2^, MB 4 J/cm^2^, and the control groups corresponding to the Raman spectra from 2800 to 3000 cm^−1^ range on days 1, 3, 7, and 14 after wounding. On day 14, this distance decreases significantly compared to days 1, 3, and 7, and the 5-ALA 4 J/cm^2^ group is the closest to the healthy skin group.

## 4. Discussion

A diabetes model has been created in mice using low doses of Streptozotocin injections. The wound healing process in the control and LDPDT groups was studied on days 1, 3, 7, and 14 after wounding. The difference between groups in wound size was found on day 14 by visual observation (see Figure 6). It was also confirmed by the wound closure percentage calculation (see Figure 7). As mentioned above, according to the Arendt–Schultz pattern, there is an interval of light dose for a positive biological response on a low-level laser therapy. In fact, LDPDT has to act similarly, with the difference that in LDPDT, there is a specific targeting to cells accumulating a PS [22]. 

From the presented results, it can be concluded that LDPDT 4 J/cm^2^ groups have a positive effect on the diabetic wound healing process, and therefore, the dose 4 J/cm^2^ is in the effective range of biological response. It is interesting that the wound healing rate was higher for the non-diabetic control group compared to LDPDT 1 J/cm^2^ diabetics groups and lower compared to LDPDT 4 J/cm^2^ groups. While the statistical difference was smaller between LDPDT 1 J/cm^2^ groups and the diabetic control, it can be said that the dose of 1 J/cm^2^ is close to the lower threshold for diabetic wound LDPDT.

The Raman spectra of wounds are dominated by the vibrational bands of skin structural proteins, amino acids, and lipids. Both position and intensity changes are observed in certain Raman spectra bands. For example, the wounds Raman spectra had significant peaks in the fingerprint spectral region of collagen.

According to Figure 11, the control group is unique in its behavior: the decrease in the 1658/1445 cm^−1^ band areas ratio indicates an impaired collagen deposition in wounds that is classified as “impaired healing wounds”. As for the LDPDT groups, they show an increasing tendency in the collagen content, and it is evidence of LDPDT’s positive role in the wound healing process. On day 14, the group 5ALA-4 J/cm^2^ shows the highest 1658/1445 cm^−1^ band areas ratio, that indicates better healing. In addition, the healthy group without diabetes also showed a tendency in the collagen content.

In the 1020–1140 cm^−1^ spectral range, there are bands associated with phosphate groups, phospholipids, and the C–C stretch of proline [57]. On day 3, these bands’ intensities in the control group wounds are higher compared to the healthy skin, as shown in Figure 12. On the other hand, these bands’ intensities in LDPDT 5-ALA groups are slightly different from the healthy skin, while the bands’ intensities of skin in LDPDT MB groups are lower compared to the healthy skin.

The difference is noticed by comparison between the LDPDT MB groups and the control. Based on this, it can be concluded that LDPDT with MB has effects on phosphate groups, phospholipids, and proline content. Phospholipids are the major targets sensitive to radicals and ROSs [62], which can be generated by LDPDT [23,63]. Lipid peroxidation occurs as the result of hydroxyl radical action, and lipid hydro-peroxides are produced that affect the structure and function of cell membrane and can be harmful to cells. They may also participate in redox reactions that can mitigate the negative effects of the injury [62]. The biological activity of phospholipids and their structural functions are drastically altered by fatty acids oxidation. Oxidation converts oxidized phospholipids (OxPLs) into markers of “modified-self” type. These markers are recognized by soluble and cell-associated receptors of innate immunity, including scavenger receptors, natural (germ line-encoded) antibodies, and C-reactive protein, thus directing removal of senescent and apoptotic cells or oxidized lipoproteins. The functional significance of OxPL accumulating during acute inflammation is not well understood yet. Products of lipid peroxidation are regarded as toxic compounds inducing tissue damage and initiating inflammation. However, under certain conditions, OxPLs also demonstrate tissue-protective and anti-inflammatory activities. OxPLs cause a variety of positive and negative inflammatory responses and, as a consequence, may have varying and context-dependent effects on the inflammatory pathway. Therefore, OxPL accumulation is a characteristic of various types of inflammatory reactions. OxPL’s inhibition of toll-like receptors and the oxidative blast may act as negative feedback, limiting activation of antibacterial mechanisms and reducing host tissue damage [64]. The nature of the effect of reduced phospholipids on wound healing requires further studies.

According to Figure 13, the 1200–1750 cm^−1^ spectral range is basically associated with the amides and CH_2_. The essential difference between the control and LDPDT groups is observed in the 1200–1300 cm^−1^ spectral range near the positions of the amide III bands, which are also characteristic bands associated with polypeptide chains, vibrations of C–N stretching and N–H bending [49]. Amide III is directly related to the structural configuration of collagen [65] associated with wound healing. On days 1, 3, 7, and 14, the wounds’ Raman spectra in the control group are lower than the LDPDT groups’ Raman spectra. On day 14, the LDPDT groups’ wound Raman spectra are close to the healthy skin Raman spectrum (in PC space). The differences in the wound Raman spectra between the control and LDPDT groups were established in the 1300–1750 cm^−1^ range, which is dominated by the CH_2_ and amide I at 1445 and 1658 cm^−1^, respectively. As previously mentioned, the 1658/1445 cm^−1^ band areas ratio is associated with collagen content. 

The 2700–3000 cm^−1^ spectral range corresponds to the CH group, which indicates relative quantities of total protein and lipid content [48]. Lipids play an important role in many cellular mechanisms relevant to the wound healing process, but not all the lipid biochemistry processes are yet well understood [66].

Diabetes is a common endogenous cause of lipid metabolism disorders [67,68,69]. Diabetes-related changes in lipid metabolism and signaling may be attributed to a significant delay in diabetic wound healing [66]. Diabetic wounds are characterized by a high inflammatory status. Leukotrienes are a major factor in the recruitment of inflammatory cells. Diabetic mice have been demonstrated to produce higher levels of leukotrienes in the skin correlating with larger non-healing wounds, excessive neutrophil migration, and uncontrolled collagen deposition [70]. The leukotrienes are lipid mediators belonging to a large family of eicosanoids. They are generated from arachidonic acid and essential eicosapentaenoic acid [71]. Arachidonic acid is present in the phospholipids of the cellular membrane. It has peaks at 2862 cm^−1^, 2886 cm^−1^, and 2923 cm^−1^ related to ν_s_(=CH_2_), ν_as_(=CH_2_), and ν_s_(=CH_3_), respectively [72]. From the above and Figure 14, it can be said that the control group is in an inflammatory state because it has a higher Raman spectra intensity associated with leukotrienes, delaying wound healing. In LDPDT groups, this factor is less compared to the control. Still, there is a need to study in more detail the role of lipids in wound healing processes.

In the initial stage of wound healing, an early provisional matrix is formed, which it is a fibrin-rich polymer with interspersed, cross-linked plasma fibronectin [73]. It can explain the decrease of the Mahalanobis distance on day 3 for LDPDT-MB groups (see Table 3). Due to overlapping wound healing stages, it is a possible reason why this behavior was not observed in the other groups. In later stages, the early provisional fibrin-rich matrix is replaced. Fibroblasts are the main cell type responsible for replacing the provisional fibrin-rich matrix with a more substantial granulation tissue [8]. Fibroblasts degrade the provisional matrix by producing MMPs and replace it with a granulation tissue rich in fibronectin and immature collagen [74]. This granulation tissue acts as a scaffold for the migration and differentiation of wound cells, regulates cell adhesion dynamics, and provides the crucial ECM template for ensuring collagen deposition [75]. Fibroblasts begin to activate in the wound site on days 2–5 after wounding as the inflammatory phase is ended and their numbers peak at one to two weeks post-wounding. By the end of the first week, fibroblasts are the main cells in the wound [76].

LDPDT plays an important role in the stimulation of fibroblast proliferation, and thus, wound healing. In some studies, an increase in fibroblasts has been seen after LDPDT of chronic wounds compared to the control group [77]. ROSs facilitated wound healing by stimulating fibroblast proliferation and migration, resulting in the development of ECM, keratinocyte growth and migration, and re-epithelialization [78]. ROSs are also known to induce transforming growth factor alpha (TGF) in fibroblasts [79]. Increased ROSs and activated TGF-signaling promote fibroblast cell proliferation and trans-differentiation into fibroblasts, as well as excessive ECM deposition and fibrosis [80]. ROSs are capable of triggering keratinocyte growth factor receptor activation and its internalization, which is an important component in epidermal regeneration [81]. The current literature indicates that photo-biomodulation can be a potent short-term way to decrease oxidative stress markers and to enhance antioxidant content [82]. LDPDT could positively affect the diabetic wound healing process, using a low concentration of 5-ALA. In both normal and diabetic cell models, it causes an increase in ROS level compared to the control group. It could suggest the positive effect of LDPDT on stimulation of dermal fibroblast proliferation caused by small amounts of ROSs activating extracellular signal-regulated kinases [22]. This explains the decrease in the Mahalanobis distance of wounds’ Raman spectra in the LDPDT groups relatively healthy skin on days 7–14. It was followed from Table 3 that the LDPDT groups, especially the 5ALA 4 J/cm^2^ group, are closer to healthy skin than the control. It is also consistent with the results of the 1658/1445 cm^−1^ band areas ratio estimations (Figure 9).

## 5. Conclusions

In summary, it was found that Raman spectroscopy is effective in monitoring biological processes such as wound healing. The effect of LDPDT on diabetic wound healing was studied by Raman spectroscopy. A method for the quantitative assessment of wound healing was developed based on the information about the chemical structure and content of the skin that can be obtained from the Raman spectra. Analysis of the changes in positions and intensities of the amide I and CH_2_ bands reveals the collagen content in the wound, which plays an important role in the wound healing process. The results indicate the positive effect of the LDPDT in diabetic wounds, where the LDPDT groups had an increased tendency for collagen content compared to the control group, and the 5ALA 4 J/cm^2^ group showed the best results. It can be concluded that a dose of 4 J/cm^2^ is in the effective range of biological response of LDPDT, while 1 J/cm^2^ is close to the lower threshold for diabetic wound LDPDT. The estimation of the Mahalanobis distance between Raman spectra in the principal component space also can be used as a quantitative assessment of the wound healing progress. 

For a future work, studies are planned in order to monitor the more complex dynamic changes that occur to the cellular and molecular elements of the wound healing process.

## Figures and Tables

**Table 1:** Weight and blood glucose levels (Mean ± StD) in control and diabetic mice groups (n = 25 per group).

		Weight (g)	Glucose (mg/dL)
a	All	30.2 ± 2.4	8.0 ± 1.7
b	Control	32.5 ± 4.3	8.7 ± 2.1
	Diabetics	31.9 ± 2.4	13.9 ± 3.9
c	Control	35.4 ± 3.2	8.9 ± 0.9
	Diabetics	30.3 ± 3.8	21.6 ± 4.7
d	Control	36.6 ± 3.1	9.5 ± 4.1
	Diabetics	31.4 ± 2.9	26.9 ± 4.0

(a) before diabetic model creation, (b) the early stage of diabetes (after 5 days of last STZ injection), (c) the later stage of diabetes (after 15 of last STZ injection), (d) after 25 days of last STZ injection.

**Table 2:** The *p*-values for all groups in comparison with healthy skin on all observation days (n = 15).

	Control	5ALA 1 J/cm^2^	5ALA 4 J/cm^2^	MB 1 J/cm^2^	MB 4 J/cm^2^
day 1	0.0041	0.0013	0.0015	0.0046	0.0082
day 3	0.0038	0.0006	0.0072	0.0013	0.0003
day 7	0.0081	0.0011	0.0007	0.0017	0.0038
day 14	0.0689	0.0002	0.0003	0.00003	0.000003

**Table 3:** Mahalanobis distances in the PC2–PC3 space between the healthy skin and other groups in the 2800–3000 cm^−1^ spectral range on various observation days.

	5ALA 1 J/cm^2^	5ALA 4 J/cm^2^	MB 1 J/cm^2^	MB 4 J/cm^2^	Control
day 1	13.1	16.2	15.7	13.8	24.8
day 3	18.3	17.1	10.8	11.7	34.9
day 7	16.5	20.4	17.5	17.8	23.2
day 14	3.8	2.9	7.6	4.1	13.0

## Data Availability

The data presented in this study are available on request from the corresponding author. The data are not publicly available due to privacy or ethical restrictions.
